# Clinical characteristics of idiopathic interstitial pneumonias with anti-Ro52/tripartite motif-containing 21 antibodies

**DOI:** 10.1038/s41598-022-15321-4

**Published:** 2022-07-01

**Authors:** Masahiro Tahara, Noriho Sakamoto, Minoru Satoh, Hiroshi Ishimoto, Hirokazu Yura, Kei Yamasaki, Takashi Kido, Yoshihisa Fujino, Tomoko Hasegawa, Shin Tanaka, Kazuhiro Yatera, Hiroshi Mukae

**Affiliations:** 1 Department of Respiratory Medicine, University of Occupational and Environmental Health, Japan, 1-1 Iseigaoka, Yahatanishi-ku, Kitakyushu, Fukuoka 807-8555 Japan; 2 Department of Respiratory Medicine, Nagasaki University Graduate School of Biomedical Sciences, Nagasaki, Japan; 3 Department of Clinical Nursing, School of Health Sciences, University of Occupational and Environmental Health, Japan, Kitakyushu, Japan; 4 Department of Environmental Epidemiology, Institute of Industrial Ecological Sciences, University of Occupational and Environmental Health, Japan, Kitakyushu, Japan; 5 Department of Human, Information and Life Sciences, School of Health Sciences, University of Occupational and Environmental Health, Japan, Kitakyushu, Japan

**Keywords:** Translational research, Rheumatology

## Abstract

Antibodies to Ro52/tripartite motif-containing 21 (TRIM21), referred to as anti-Ro52, are found in patients diagnosed with diverse systemic autoimmune rheumatic disease and associated with interstitial lung diseases. However, little is known about the clinical characteristics of anti-Ro52 in patients with idiopathic interstitial pneumonias (IIPs). We aimed to analyze the prevalence, co-existent autoantibodies, and clinical characteristics of anti-Ro52 in patients with IIP. The study enrolled 288 patients diagnosed with IIP. Clinical, laboratory and radiographic findings of IIP patients were compared between anti-Ro52 positives and negatives. Anti-Ro52 (20/288; 6.9%), anti-ARS (18/288; 6.3%), and anti-Ro60/SS-A (16/288; 5.6%) were the most common autoantibodies detected in IIP patients. Among 20 IIP patients who had anti-Ro52, anti-ARS was present in 8 (40%) patients. The criteria for interstitial pneumonia with autoimmune features (IPAF) were significantly better fulfilled by patients with anti-Ro52 than those without (P = 0.001). Meeting serological domain (P < 0.001) and Raynaud’s phenomenon (P = 0.009) were significantly more common in the anti-Ro52-positive patients. Anti-Ro52-positive IIP patients have clinical features consistent with IPAF. Anti-Ro52 may have an important role in detecting the autoimmune phenotype in IIP patients.

## Introduction

Idiopathic interstitial pneumonias (IIPs) are diffuse fibrotic lung disorders that exclude known causes of interstitial lung diseases (ILDs) such as systemic autoimmune rheumatic disease (SARD), environmental exposure, and medication toxicity^1,2^. Patients with IIP who have autoimmune features, but do not meet established diagnostic/classification criteria of SARD^3,4^ are categorized as “interstitial pneumonia with autoimmune features (IPAF)”^5^. Autoantibodies such as anti-aminoacyl-tRNA synthetases (anti-ARS) have proved clinically significant in diagnosis, treatment, and prediction of prognosis of IIP patients fulfilling the IPAF criteria^5–8^.

Ro52/tripartite motif-containing 21 (TRIM21), an E3 ubiquitin ligase involved in ubiquitination, plays a prominent regulatory role in inflammation, apoptosis, and oxidative stress^9–11^. Anti-Ro52/TRIM21 antibodies (anti-Ro52) are commonly detected in the sera of patients diagnosed with different types of SARD, including Sjögren's syndrome^12^, polymyositis/dermatomyositis (PM/DM)^13,14^, systemic sclerosis (SSc)^15,16^, and systemic lupus erythematosus (SLE)^17^. Co-existence of anti-Ro52 and anti-ARS (particularly anti-Jo-1 and anti-PL-7) is common in patients with PM/DM^14,18,19^. Anti-Ro52 is associated with the presence of ILD in SSc, PM/DM, and mixed connective tissue disease (MCTD)^13,15,16,20^. One study in PM/DM reported higher prevalence of ILD in patients with anti-Ro52 than without, however, it might be due to an association of anti-Ro52 and anti-ARS^14^. Another study on patients with anti-Ro52-positive ILD reported the absence of an established diagnosis of SARD in the majority (78%) of patients while nearly half (49.3%) fulfilled the IPAF criteria^21^. Further studies are warranted to explore the prevalence, co-existent autoantibodies, and clinical characteristics associated with the presence of anti-Ro52 in IIP patients.

This study aims to analyze the clinical significance of anti-Ro52 in patients with IIPs and the associated clinical and immunological characteristics. The findings from this study may contribute to more accurate classification of IIP.

## Results

### Autoantibodies in sera of patients with IIP

Of the 288 IIP patients enrolled in the study, ELISA revealed the presence of anti-Ro52 in the sera of 20 patients (6.9%) (Table 1). ANA and RF positivity in the serological domain of the IPAF were as follows; ANA titer ≥ 1:320, diffuse, speckled, homogeneous patterns (n = 20; 7.1%), ANA any titer, nucleolar or centromere patterns (n = 14; 5.0%), RF ≥ 2 × upper limit of normal (n = 24; 9.4%). Anti-ARS was detected in 18 patients (6.3%), including autoantibodies to Jo-1 (n = 5; 1.7%), PL-7 (n = 2; 0.7%), PL-12 (n = 1; 0.3%), EJ (n = 4; 1.4%), OJ (n = 1; 0.3%), and KS (n = 5; 1.7%) (Table 1). In addition, autoantibodies to the following antigens were detected; Ro60/SS-A (n = 16; 5.6%), La/SS-B (n = 1; 0.3%), CCP (n = 9; 5.0%), double stranded DNA (n = 6; 3.3%), U1RNP (n = 2; 0.7%), topoisomerase I (Scl-70) (n = 1; 0.3%), MDA5 (n = 1; 0.3%), TIF1β (n = 2; 0.7%), CENP-A (n = 3; 1.0%), CENP-B (n = 2; 0.7%), and Anti-RNA Polymerase III (n = 1; 0.3%) (Table 1).

**Table 1:** Prevalence of autoantibodies in idiopathic interstitial pneumonitis (IIP) patients.

Autoantibodies	Prevalence n (%)	Numbers tested n
Anti-Ro52	20 (6.9)	288
ANA titer ≥ 1:320, diffuse, speckled, or homogeneous patterns	20 (7.1)	280
ANA any titer, nucleolar or centromere patterns	14 (5.0)	280
Rheumatoid factor ≥ 2 × upper limit of normal	24 (9.4)	255
**Anti-ARS**	18 (6.3)	288
Anti-Jo-1	5 (1.7)	288
Anti-PL-7	2 (0.7)	288
Anti-PL-12	1 (0.3)	288
Anti-EJ	4 (1.4)	288
Anti-OJ	1 (0.3)	288
Anti-KS	5 (1.7)	288
Anti-Ro60/SS-A	16 (5.6)	288
Anti-La/SS-B	1 (0.3)	288
Anti-CCP	9 (5.0)	180
Anti-double stranded DNA	6 (3.3)	183
Anti-U1RNP	2 (0.7)	288
Anti-Sm	0 (0)	288
Anti-topoisomerase I (Scl-70)	1 (0.3)	288
Anti-PM-Scl	0 (0)	288
Anti-MDA5	1 (0.3)	288
Anti-TIF1β	2 (0.7)	288
Anti-CENP-A	3 (1.0)	288
Anti-CENP-B	2 (0.7)	288
Anti-RNA polymerase III	1 (0.3)	288

Data presented as frequencies (%).

Ro52: Ro52/tripartite motif-containing 21; ANA: anti-nuclear antibody; ARS: aminoacyl-tRNA synthetases; CCP: cyclic citrullinated peptide; MDA5: melanoma differentiation-associated gene 5; TIF1: transcriptional intermediary factor 1; CENP: centromere protein.

### Autoantibodies co-existing with anti-Ro52

Co-existence of anti-Ro52 with other autoantibodies was analyzed (Table 2). The presence of ANA (ANA titer ≥ 1:320, diffuse, speckled, or homogeneous patterns, 20% vs. 6.2%, P = 0.04), anti-ARS (40% vs. 3.7%, P < 0.001), and anti-Ro60/SS-A (30% vs. 3.7%, P < 0.001) was significantly more common in anti-Ro52-positive than in anti-Ro52-negative patients (Table 2). Among eight patients positive for both anti-ARS and anti-Ro52, three were positive for anti-Jo-1, two for anti-KS, and one each for anti-PL-7, anti-PL-12, and anti-EJ.

**Table 2:** Antibodies co-existing with anti-Ro52/tripartite motif-containing 21 antibodies (anti-Ro52).

Subjects	Anti-Ro52 positive	Anti-Ro52 negative	*P* value
	n = 20 n (%)	n = 268 n (%)	
ANA titer ≥ 1:320, diffuse, speckled, or homogeneous patterns	4 (20)	16 (6.2)	0.04
ANA any titer, nucleolar or centromere patterns	3 (15)	11 (4.2)	0.07
Rheumatoid factor ≥ 2 × upper limit of normal	3 (20)	21 (8.8)	0.16
**Anti-ARS**	8 (40)	10 (3.7)	< 0.001
Anti-Jo-1	3 (15)	2 (0.7)	0.003
Anti-PL-7	1 (5)	1 (0.4)	0.13
Anti-PL-12	1 (5)	0 (0)	0.07
Anti-EJ	1 (5)	3 (1.1)	0.25
Anti-OJ	0 (0)	1 (0.4)	0.93
Anti-KS	2 (10)	3 (1.1)	0.04
Anti-Ro60/SS-A	6 (30)	10 (3.7)	< 0.001
Anti-La/SS-B	0 (0)	1 (0.4)	0.93
Anti-CCP	0 (0)	9 (5.4)	0.47
Anti-double stranded DNA	0 (0)	6 (3.6)	0.57
Anti-U1RNP	1 (5)	1 (0.4)	0.13
Anti-Sm	0 (0)	0 (0)	–
Anti-topoisomerase I (Scl-70)	0 (0)	1 (0.4)	0.93
Anti-PM-Scl	0 (0)	0 (0)	–
Anti-MDA5	0 (0)	1 (0.4)	0.93
Anti-TIF1β	0 (0)	2 (0.7)	0.87
Anti-CENP-A	0 (0)	3 (1.1)	0.81
Anti-CENP-B	0 (0)	3 (1.1)	0.87
Anti-RNA polymerase III	0 (0)	1 (0.4)	0.93

Data presented as frequencies (%).

Ro52: Ro52/tripartite motif-containing 21; ANA: anti-nuclear antibody; ARS: aminoacyl-tRNA synthetases; CCP: cyclic citrullinated peptide; MDA5: melanoma differentiation-associated gene 5; TIF1: transcriptional intermediary factor 1; CENP: centromere protein.

*P* value: anti-Ro52 positive vs. anti-Ro52 negative.

### Anti-Ro52: patient characteristics and clinical course

**Figure 1:**
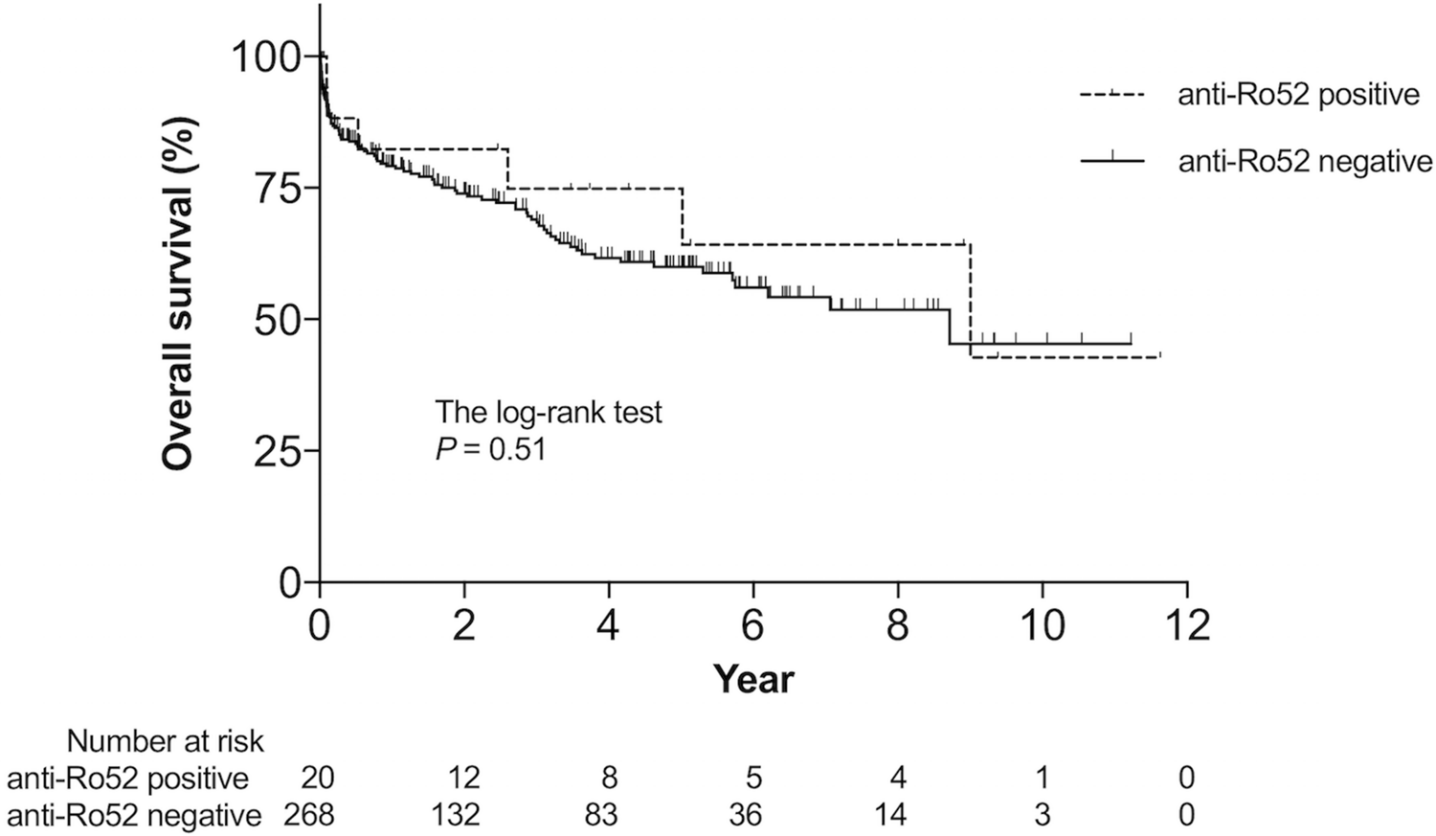
Kaplan–Meier survival curves of idiopathic interstitial pneumonia (IIP) patients with/without anti-Ro52/tripartite motif-containing 21 antibodies (anti-Ro52). Kaplan–Meier curves representing the survival rate of IIP patients in the presence (dotted line; n = 20) and absence (dashed line; n = 268) of serum anti-Ro52. Statistically relevant difference was not observed between the analyzed groups (log-rank *P* = 0.51).

Clinical characteristics of anti-Ro52-positive vs. -negative patients are summarized in Table 3. Anti-Ro52-positive patients frequently met the IPAF criteria (50% vs. 17%, P = 0.001), had the clinical domain of IPAF criteria (20% vs. 8%, P = 0.09), and showed a greater percentage of fulfillment of serological domain (75% vs. 26%, P < 0.001), but had similar prevalence of morphological domain (40% vs. 44%) compared to anti-Ro52-negative patients. Raynaud's phenomenon was significantly more common in anti-Ro52-positive than in anti-Ro52-negative patients (15% vs. 2%, P = 0.009). Laboratory findings indicated significantly higher levels of serum Krebs von den Lungen-6 (KL-6) in anti-Ro52-positive than in anti-Ro52-negative patients (1258 U/mL vs. 858 U/mL, P = 0.01) (Table 4). HRCT analyses revealed more frequent lower distribution (90% vs. 69%, P = 0.03) and less frequent ground-glass attenuations (45% vs. 72%, P = 0.02) in anti-Ro52-positive than in anti-Ro52-negative patients (Table 5). Significant differences were not detected in HRCT patterns in the presence or absence of serum anti-Ro52 in IIP patients. However, OP and DAD were seen exclusively in anti-Ro52-negative patients. Patient characteristics and details of each domain are shown in Supplementary Tables S1 and S2. There were no patients who developed and fulfilled the classification/diagnostic criteria of SARD during a median observation period of 771 days in this cohort study. Kaplan–Meier curves showed no significant difference in the overall survival rate between patients with and without serum anti-Ro52 (log-rank P = 0.51) (Fig. 1).

**Table 3:** Clinical characteristics of patients in the presence or absence of anti-Ro52/tripartite motif-containing 21 antibodies (anti-Ro52).

Subjects	All	Anti-Ro52 positive	Anti-Ro52 negative	*P* value
	n = 288	n = 20	n = 268	
Age (years)	69.5 [63–75]	67.5 [63–74]	70 [63–75]	0.35
Male, n (%)	193 (67)	11 (55)	182 (68)	0.17
Smoking (pack-years)	22 [0–48]	19 [0–42]	23 [0–49]	0.79
**Fulfilled IPAF criteria, n (%)**	55 (19)	10 (50)	45 (17)	0.001
Clinical domain, n (%)	26 (9)	4 (20)	22 (8)	0.09
Serological domain, n (%)	86 (30)	15 (75)	71 (26)	< 0.001
Morphological domain, n (%)	127 (44)	8 (40)	119 (44)	0.44
**Respiratory symptoms**				
Cough^#^, n (%)	164 (59)	12 (63)	152 (59)	0.47
Sputum^¶^, n (%)	62 (23)	2 (11)	60 (24)	0.15
Dyspnea^+^, n (%)	177 (67)	15 (83)	162 (66)	0.10
**Clinical symptoms related to SARD**				
Mechanic's hands^§^, n (%)	3 (1)	1 (5)	2 (1)	0.20
Distal digital tip ulceration^ƒ^, n (%)	2 (1)	0 (0)	2 (1)	0.87
Inflammatory arthritis^ƒ^, n (%)	15 (5)	1 (5)	14 (5)	0.72
Palmar telangiectasia^§^, n (%)	0 (0)	0 (0)	0 (0)	–
Raynaud's phenomenon^§^, n (%)	7 (2)	3 (15)	4 (2)	0.009
Unexplained digital edema^§^, n (%)	2 (1)	0 (0)	2 (1)	0.864
Gottron's sign^ƒ^, n (%)	0 (0)	0 (0)	0 (0)	–
Muscle weakness^##^, n (%)	2 (1)	0 (0)	2 (1)	0.87
Weight loss^¶¶^, n (%)	15 (5)	1 (5)	14 (5)	0.71
Dry mouth or dry eye^ƒ^, n (%)	7 (2)	0 (0)	7 (3)	0.60
Dysphagia^ƒ^, n (%)	6 (2)	0 (0)	6 (2)	0.65
Gastroesophageal reflux disease^++^, n (%)	23 (36)	1 (25)	22 (37)	0.55

Data presented as median [interquartile range] or frequencies (%).

n = 288, unless otherwise stated; ^#^n = 276; ^¶^n = 273; + n = 263; ^§^n = 285; ^ƒ^n = 286; ^##^n = 287; ^¶¶^n = 282; ^++^n = 64.

Ro52: Ro52/tripartite motif-containing 21; IPAF: interstitial pneumonia with autoimmune features; SARD: systemic autoimmune rheumatic diseases.

*P* value: anti-Ro52 positive vs. anti-Ro52 negative.

**Table 4:** Tests and findings of patients with/without anti-Ro52/tripartite motif-containing 21 antibodies (anti-Ro52).

Subjects	All	Anti-Ro52 positive	Anti-Ro52 negative	*P* value
	n = 288	n = 20	n = 268	
**Laboratory findings**				
CRP (mg/dL)^#^	0.48 [0.12–2.66]	0.30 [0.17–1.73]	0.49 [0.12–2.7]	0.93
LDH (IU/L)^¶^	225 [192–288]	226 [187–291]	225 [193–288]	0.54
CK (IU/L)^+^	74 [51–114]	77 [55–126]	73 [51–113]	0.42
KL-6 (U/mL)^§^	878 [491–1554]	1258 [807–2604]	858 [476–1462]	0.01
SP-A (ng/mL)^ƒ^	73 [49–107]	87 [72–109]	72 [47–107]	0.36
SP-D (ng/mL)^##^	220 [118–340]	180 [121–335]	220 [118–341]	0.91
**Pulmonary function tests**^**¶¶**^				
VC (% predicted)	79 [64–94]	76 [63–85]	80 [64–95]	0.36
FEV_1_/FEV (% predicted)	82 [76–88]	81 [78–86]	82 [76–88]	0.78
DL_CO_ (% predicted)	59 [42–74]	63 [41–68]	58 [42–74]	0.76
**Bronchoalveolar lavage fluid**^**++**^				
TCC (× 10^5^/mL)	2.8 [1.8–4.5]	2.9 [1.7–4.9]	2.8 [1.8–4.5]	0.84
Macrophages (%)	69.1 [50.3–80.6]	76.9 [62.0–78.9]	68.7 [50.0–80.7]	0.43
Lymphocytes (%)	12.7 [6.5–28.1]	10.8 [6.2–18.2]	12.8 [6.5–18.1]	0.39
Neutrophils (%)	6.3 [2.9–13.5]	4.5 [2.8–10.3]	6.6 [2.9–13.6]	0.64
Eosinophils (%)	3.1 [1.1–6.2]	3.3 [1.3–4.3]	3.1 [1.1–6.3]	0.80
CD4/CD8	1.7 [0.8–2.7]	1.4 [0.4–3.5]	1.7 [0.8–2.7]	0.60

Data presented as median [interquartile range] or frequencies (%).

n = 288, unless otherwise stated; ^#^n = 279; ^¶^n = 278; ^+^n = 229; ^§^n = 260; ^ƒ^n = 137; ^##^n = 210; ^¶¶^n = 209; ^++^n = 218.

Ro52: Ro52/tripartite motif-containing 21; CRP: C-reactive protein; LDH: lactate dehydrogenase; CK: creatine kinase; KL-6: Krebs von den Lungen-6; SP-A: surfactant protein-A; SP-D: surfactant protein-D; VC: vital capacity; FEV_1_: forced expiratory volume in one second; DL_CO_: diffusing capacity of the lung for carbon monoxide; TCC: total cell counts; CD: cluster of differentiation.

*P* value: anti-Ro52 positive vs. anti-Ro52 negative.

**Table 5:** Results and patterns of patients with/without anti-Ro52/tripartite motif-containing 21 antibodies (anti-Ro52).

Subjects	All	Anti-Ro52 positive	Anti-Ro52 negative	*P* value
	n = 288 n (%)	n = 20 n (%)	n = 268 n (%)	
**HRCT findings**				
Volume loss	201 (70)	17 (85)	184 (69)	0.10
Lower distribution	201 (70)	18 (90)	183 (69)	0.03
Subpleural distribution	200 (69)	13 (65)	187 (70)	0.41
Peribronchial distribution	40 (14)	3 (15)	37 (14)	0.55
Reticular shadow	241 (84)	18 (90)	223 (84)	0.34
Honeycombing	110 (38)	10 (50)	100 (38)	0.19
Traction bronchiectasis	228 (79)	19 (95)	209 (79)	0.052
Ground-glass attenuation	200 (69)	9 (45)	191 (72)	0.02
Consolidation	88 (31)	4 (20)	84 (32)	0.21
Thickening of BVB	7 (2)	1 (5)	6 (2)	0.40
Small nodules (φ < 5 mm)	17 (6)	0 (0)	17 (6)	0.28
Nodules (φ > 5 mm)	13 (5)	1 (5)	12 (5)	0.62
Pleural effusion	14 (5)	0 (0)	14 (5)	0.36
**HRCT pattern**				
UIP	120 (42)	9 (45)	111 (42)	0.46
NSIP	91 (32)	9 (45)	82 (31)	0.14
OP	31 (11)	0 (0)	31 (12)	0.09
DAD	11 (4)	0 (0)	11 (4)	0.45
Others	35 (12)	2 (10)	33 (12)	0.55

Data presented as frequencies (%).

Ro52: Ro52/tripartite motif-containing 21; HRCT: high-resolution computed tomography; BVB: bronchovascular bundles; UIP: usual interstitial pneumonia; NSIP: nonspecific interstitial pneumonia; OP: organizing pneumonia; DAD: diffuse alveolar damage.

*P* value: anti-Ro52 positive vs. anti-Ro52 negative.

### Anti-Ro52 in anti-ARS-positive cases: patient characteristics and survival

The clinical characteristics and survival of anti-Ro52-positive vs. -negative patients who were also positive for anti-ARS are summarized in Supplementary Tables S3–5 and Supplementary Fig. S1. Clinical characteristics and Kaplan–Meier curves showed no significant difference in anti-ARS-positive patients with and without serum anti-Ro52.

## Discussion

This is the first study investigating the frequency of serum anti-Ro52 antibodies in unselected patients with IIP. Similar to the prevalence of anti-ARS (6.3%), anti-Ro52 was detected in 6.9% of patients with IIP. Presence of serum anti-Ro52 was significantly associated with fulfillment of IPAF criteria, particularly with respect to the serological domain and Raynaud’s phenomenon, in IIP patients.

Anti-Ro52 is mostly present in patients with different types of SARD^22^, as seen in nearly half of the patients with Sjögren's syndrome^12^, SSc^15,16^, and SLE^17^ and 20–30% of patients with PM/DM^13,14^, In this study, the prevalence of anti-Ro52 (6.9%) in IIP was lower than in SARD but higher than in healthy individuals (< 0.2–1%)^23^. In addition, as in patients with PM/DM^14,18,19^, anti-Ro52 frequently co-existed with anti-ARS. Among the anti-ARS that co-existed with anti-Ro52, anti-Jo-1 found in three cases was the most common, in addition to anti-PL-7, anti-PL-12, anti-EJ, and anti-KS.

IPAF criteria (P = 0.001) related to the serological domain (P < 0.001) were more frequently fulfilled by anti-Ro52-positive (50%) than anti-Ro52-negative patients (17%) in our IIP cohort. A previous retrospective study showed that 49.3% of the ILD patients who had anti-Ro52 met the IPAF criteria, similar to our result^21^. Anti-Ro52-positive patients could be negative in immunofluorescence ANA tests, however, anti-Ro52 was associated with IPAF serological domain, indicating that it frequently coexists with the other autoantibodies included in the IPAF serological domain (Table 2). Co-existence of anti-Ro52 with other specific autoantibodies in various SARD have been reported^14,15,18^. Although anti-Ro52 is not specific for a particular type of SARD diagnosis, a 14-fold increased risk of developing SARD was reported in IIP patients who met the IPAF criteria^24^. Thus, presence of anti-Ro52 might be considered as a useful clinical diagnostic tool for the early detection of SARDs in patients with IIP who pose a higher risk of developing in the future.

IPAF criteria are used for the identification of a subset of IIP patients exhibiting autoimmune features but lacking a definitive diagnosis of SARD^5^ The ATS/ERS task force has suggested the need for further validation and revision of IPAF criteria^5^. Accordingly, there has been a proposal for the inclusion of several myositis-specific antibodies (anti-NXP-2, anti-TIF1γ) in the IPAF criteria^25^. In contrast, anti-double stranded DNA, anti-Sm, anti-topoisomerase I (Scl-70), and anti-MDA5 are disease-specific diagnostic antibodies that have a proven link to the diagnosis of SLE^26^, SSc^27^, and clinically amyopathic DM (CADM)^28^. These disease-specific marker antibodies are produced prior to the clinical manifestation of the associated SARD and the association of IPAF with these antibodies might be an indication of early stage SARD. The appropriateness of the inclusion of these antibodies in the IPAF criteria is controversial.

Some commercial assays separately measure antibodies to Ro60 and Ro52, while other anti-Ro/SS-A immunoassays use a mixture of Ro60 and Ro52 as antigen. However, recent literature indicates that Ro60 and Ro52/TRM21 are unrelated molecule and Ro52/TRIM21 is not a part of Ro60/SS-A-hYRNAs complex^29–31^. Thus, separate measurement of anti-Ro52 and anti-Ro60/SS-A is recommended because of their biochemical and immunological differences^32^. In our study, 70% (14 of 20) of anti-Ro52 positive were negative for anti-Ro60/SS-A. It remains unclear whether the definition of “anti-Ro (SS-A)” in the IPAF criteria meant a mixture of anti-Ro52 and anti-Ro60/SS-A or anti-Ro60/SS-A alone; therefore, our findings suggested that the definition of “anti-Ro (SS-A)” in the IPAF criteria should be clarified and testing anti-Ro52/TRIM21 and anti-Ro60/SS-A separately to identify the autoimmune phenotype in IIP patients.

The frequency of Raynaud's phenomenon was significantly higher in anti-Ro52-positive patients than in anti-Ro52-negative patients in our IIP cohort (P = 0.009) (Table 3). Nearly half of the IPAF patients exhibit at least one clinical domain with Raynaud's phenomenon as the most common symptom^33,34^. In this study, all three patients with anti-Ro52 who had Raynaud's phenomenon were classified as IPAF (Supplementary Tables S1 and S2). Thus, testing for serum anti-Ro52 might be helpful in classifying IIP patients with Raynaud’s phenomenon as those meeting the IPAF criteria. Raynaud's phenomenon is associated with underlying or future development of SARD^35^ but is not considered a predictor for its prognosis or development in IPAF patients^33,34^ probably due to the low prevalence and short follow-up periods. Thus, the clinical significance of Raynaud's phenomenon in IPAF patients remains controversial.

Patients with anti-Ro52 have a higher frequency of rapidly progressive ILD and a higher rate of mortality than those without anti-Ro52 in SARD^13,15,16,20^. Herein, presence of anti-Ro52 was not significantly associated with overall mortality, possibly due to the heterogeneity of IIPs and the limited number of patients.

Patients with anti-ARS are associated with a unique subset characterized by clinical features, including ILDs, called anti-synthetase syndrome (AS), and several criteria of AS have been proposed^36,37^. However, AS is a “syndrome” developed for research settings, and its concept is still controversial. Recent research has reported the heterogeneity related to the prognosis and response to treatment of IIP patients with anti-ARS, wherein, certain patients were refractory to treatment with poor prognosis, while others responded well^7,38^. Patients with PM/DM positive for both anti-Ro52 and anti-ARS had severe myositis and joint impairment with a higher prevalence of ILD^14,16^. In this study, among 18 anti-ARS-positive patients, significant differences were not seen in symptoms, characteristics (Supplementary Tables S3–5), and prognosis (Supplementary Fig. S1) related to SARD, between anti-Ro52-positive and -negative patients. However, these findings might considerably be affected by the small number of IIP patients with anti-ARS and further research is thus required.

Several limitations of this study are acknowledged. First, this study was a retrospective study with variable follow-up intervals and periods. Second, the sample size was relatively small and comprised only of Japanese individuals from two university hospitals. Third, possible missed signs and symptoms in the clinical domain of IPAF criteria may have resulted in inaccurate IPAF diagnoses because our cohort included patients enrolled before IPAF criteria was proposed. However, we routinely consulted ILD patients with rheumatologists and requested appropriate evaluations to exclude the presence of collagen vascular diseases. Fourth, although none of the patients with IIP developed any autoimmune diseases during the follow-up period, the observation period was short. It is possible that some might develop SARD in the future because ILD could precede the development of SARD in certain patients^24^.

In conclusion, the fulfillment of IPAF criteria and presence of Raynaud's phenomenon were more frequently seen in the presence than in the absence of anti-Ro52 in patients with IIP. Our findings may suggest that testing for anti-Ro52 help to identify the autoimmune phenotype and predict the development of SARD in IIP patients. Further prospective studies on a large cohort are needed to elucidate the clinical significance of anti-Ro52 in patients with IIP.

## Methods

### Study participants

A two-center retrospective cohort study was conducted by the Department of Respiratory Medicine, Nagasaki University Graduate School of Biomedical Sciences, Nagasaki, Japan, and the Department of Respiratory Medicine, University of Occupational and Environmental Health, Kitakyushu, Japan. Patients with IIP were enrolled in the study between March 2007 and October 2016 (n = 288). At the first visit, IIP was diagnosed based on clinical, laboratory, and radiological findings as per the definition of the American Thoracic Society/European Respiratory Society (ATS/ERS) international multidisciplinary consensus classification^1,2^. The study was conducted in accordance with the amended Declaration of Helsinki. The Institutional Review Board of the Nagasaki University Hospital, Nagasaki, Japan (Approval No: 16042517), and the University of Occupational and Environmental Health, (Approval No: H27-238) approved the protocol. Informed consent was obtained from all subjects. Observation and follow-up of each patient was conducted on an annual basis and was censored on April 30, 2020. Patients who were lost to follow-up were censored at the date of last contact/follow-up and those alive as on April 30, 2020, were censored for overall survival analyses.

### Detection of serum autoantibodies

Serum samples of patients were obtained during their first visit and stored at − 20 °C until further use. For the analyses of autoantibodies, ^35^S-methionine radiolabeled K562 cell extract was immunoprecipitated with IgG purified from 8 µL of human serum samples. The immunoprecipitated proteins were electrophoresed on sodium dodecyl sulfate–polyacrylamide gel electrophoresis (SDS-PAGE) as described previously^39^. Briefly, cells were labeled with ^35^S-methionine and cysteine, lysed in 0.5 M NaCl, 2 mM ethylenediaminetetraacetic acid (EDTA), 50 mM Tris (pH 7.5), 0.3% octylphenyl polyethylene glycol (IGEPAL CA-630) buffer containing 0.5 mM phenylmethylsulfonyl fluoride and 0.3 trypsin inhibitory units (TIU)/mL aprotinin, and immunoprecipitated using protein-A-Sepharose beads coated with IgG. Immunoprecipitates were then washed with 0.5 M NaCl-NET/IGEPAL CA-630 and analyzed by SDS-PAGE and autoradiography. The specificity of autoantibodies was confirmed by the use of human reference sera^39^. Antibodies to Ro52/TRIM21, histidyl-tRNA synthetase (Jo-1), and melanoma differentiation-associated protein 5 (MDA5) were tested by enzyme-linked immunosorbent assay (ELISA) as described previously^39^. All recombinant proteins were purchased from Diarect (Freiburg, Germany). Briefly, 96-well microtiter plates (Immobilizer Amino; Nunc Naperville, IL, USA) were coated with 0.5 μg/ml of recombinant protein and blocked with 0.5% bovine serum albumin (BSA)-NET/ IGEPAL CA-630 for 1 h at room temperature. Patients’ sera (1:250) and alkaline phosphatase-conjugated goat anti-human IgG (1:1000; γ-chain specific; Jackson Immunoresearch, Hershey, PA, USA) diluted in 0.5% BSA-NET/ IGEPAL CA-630 were used as the sample and secondary antibodies, respectively. A standard curve was generated using serial 1:5 dilutions of a high-titer prototype serum. Optical density of samples measured at 405 nm was converted into units based on the standard curve.

Anti-nuclear antibodies (ANA) and rheumatoid factor (RF) positivity was defined in accordance with the classification criteria of IPAF^5^: ANA titer ≥ 1:320, diffuse, speckled, homogeneous patterns; ANA any titer, nucleolar or centromere patterns; RF ≥ 2 × upper limit of normal.

### Clinical data collection and analyses

Demographic data, clinical information, results of laboratory and pulmonary function tests, and analyses of bronchoalveolar lavage fluid were obtained from medical records. Physical findings were confirmed by rheumatologists when appropriate. Classification criteria of IPAF were based on the 2015 ERS/ATS Task Force research statement^5^. Although patient data (n = 281) used in this study partially overlapped with a retrospective study published earlier^6^, our research data related to anti-Ro52 are unique.

### Radiographic evaluation

At the first visit, patients were examined by high-resolution chest computed tomography (HRCT), and evaluated independently by two pulmonologists (N. S. and H. I.) for volume loss, distribution and presence of reticular shadows, honeycombing, traction bronchiectasis, ground-glass attenuation, consolidations, thickening of bronchovascular bundles, small nodules (φ < 5 mm), nodules (φ > 5 mm), and pleural effusion. According to the international IIPs classification^2^, HRCT patterns were classified into usual interstitial pneumonia (UIP), nonspecific interstitial pneumonia (NSIP), organizing pneumonia (OP), and diffuse alveolar damage (DAD).

### Statistical analyses

Data are presented as the median [interquartile range] or frequency (%). Fisher’s exact test was used to compare categorical variables. Comparisons between groups were made using the Mann–Whitney U test. Survival analyses were performed using the Kaplan–Meier method and the log-rank test. All analyses were conducted at a significance level of α = 0.05. All statistical analyses were performed using the STATA 16.1 software (StataCorp, College Station, TX, USA).

### Ethics declarations

The Institutional Review Board of the Nagasaki University Hospital, Nagasaki, Japan (Approval No: 16042517), and the University of Occupational and Environmental Health, (Approval No: H27-238) approved the protocol. Informed consent was obtained from all subjects.

## Supplementary Information

Supplementary Information.

## Data Availability

The datasets used for the current study are available from the corresponding author on reasonable request.
