# Supplementary material for: Clinical characteristics of idiopathic interstitial pneumonias with anti-Ro52/tripartite motif-containing 21 antibodies
Source: Sci Rep. 2022 Jul 1;12:11122. doi: 10.1038/s41598-022-15321-4 (PMC9249750; doi:10.1038/s41598-022-15321-4)
Supplement: Supplementary file 1 — Supplementary Information. [file 41598_2022_15321_MOESM1_ESM.doc]

**Supplementary Information**

**Serum Anti-Ro52/Tripartite Motif-containing 21, A Novel Criterion of Interstitial Pneumonia with Autoimmune Features**

*Masahiro Tahara^a^; Noriho Sakamoto^b^; Minoru Satoh^c^; Hiroshi Ishimoto^b^; Hirokazu Yura^b^; Kei Yamasaki^a^; Takashi Kido^a,b^; Yoshihisa Fujino^d^; Tomoko Hasegawa^c^; Shin Tanaka^e^; Kazuhiro Yatera^a*^; Hiroshi Mukae^b^*

^a^Department of Respiratory Medicine, University of Occupational and Environmental Health, Japan, Kitakyushu, Japan

^b^Department of Respiratory Medicine, Nagasaki University Graduate School of Biomedical Sciences, Nagasaki, Japan

^c^Department of Clinical Nursing, School of Health Sciences, University of Occupational and Environmental Health, Japan, Kitakyushu, Japan

^d^Department of Environmental Epidemiology, Institute of Industrial Ecological Sciences, University of Occupational and Environmental Health, Japan, Kitakyushu, Japan

^e^Department of Human, Information and Life Sciences, School of Health Sciences, University of Occupational and Environmental Health, Japan, Kitakyushu, Japan

***Corresponding author:**

Kazuhiro Yatera, M.D., Ph.D.

Department of Respiratory Medicine, University of Occupational and Environmental Health, Japan, 1-1 Iseigaoka, Yahatanishi-ku, Kitakyushu, Fukuoka 807-8555, Japan

Email: yatera@med.uoeh-u.ac.jp

**Supplementary Figure S1. Kaplan–Meier curves of survival of anti-aminoacyl-tRNA synthetases antibodies (anti-ARS) positive idiopathic interstitial pneumonia (IIP) patients in the presence or absence of anti-Ro52/tripartite motif-containing 21 (TRIM21) antibodies (anti-Ro52).**

Kaplan–Meier curves representing the survival rate of anti-ARS positive IIP patients in the presence (dotted line; n = 8) and absence (dashed line; n = 10) of serum anti-Ro52. Statistically relevant difference was not observed between the analyzed groups (log-rank *P* = 0.30).

**Supplementary Table S1. Characteristics and each domain of idiopathic interstitial pneumonia (IIP) patients who fulfilled interstitial pneumonia with autoimmune features (IPAF) criteria in the presence of anti-Ro52/tripartite motif-containing 21 (TRIM21) antibodies (anti-Ro52)**

| Case | |  | 1 | 2 | 3 | 4 | 5 | 6 | 7 | 8 | 9 | 10 |
| --- | --- | --- | --- | --- | --- | --- | --- | --- | --- | --- | --- | --- |
| Age (years) | | | 62 | 78 | 64 | 69 | 68 | 65 | 65 | 31 | 74 | 55 |
| Sex | |  | F | F | F | F | M | F | F | F | M | M |
| Clinical Domain | | | – | – | – | + | + | – | + | – | – | + |
|  | 1. Mechanic's hands | | – | – | – | – | – | – | – | – | – | + |
|  | 2. Distal digital tip ulceration | | – | – | – | – | – | – | – | – | – | – |
|  | 3. Inflammatory arthritis | | – | – | – | – | – | – | – | – | – | + |
|  | 4. Palmar telangiectasia | | – | – | – | – | – | – | – | – | – | – |
|  | 5. Raynaud's phenomenon | | – | – | – | + | + | – | + | – | – | – |
|  | 6. Unexplained digital oedema | | – | – | – | – | – | – | – | – | – | – |
|  | 7. Gottron's sign | | – | – | – | – | – | – | – | – | – | – |
| Serological Domain | | | + | + | + | – | + | + | + | + | + | + |
|  | 1. ANA positive | | + | + | – | – | + | – | – | – | – | – |
|  | ANA titer ≥ 1:320, diffuse, speckled, or homogeneous patterns | | – | – | – | – | + | – | – | – | – | – |
|  | ANA any titer, nucleolar or centromere patterns | | + | + | – | – | + | – | – | – | – | – |
|  | 2. Rheumatoid factor ≥ 2× upper limit of normal | | – | – | – | – | – | n/a | n/a | + | – | n/a |
|  | 3. Anti-CCP | | – | – | – | – | – | – | n/a | n/a | – | – |
|  | 4. Anti-double stranded DNA | | – | – | – | – | – | – | n/a | – | – | – |
|  | 5. Anti-Ro60/SS-A | | – | + | + | – | – | – | – | – | – | – |
|  | 6. Anti-La/SS-B | | – | – | – | – | – | – | – | – | – | – |
|  | 7. Anti-U1RNP | | – | – | – | – | – | – | – | – | – | – |
|  | 8. Anti-Sm | | – | – | – | – | – | – | – | – | – | – |
|  | 9. Anti-topoisomerase I (Scl-70) | | – | – | – | – | – | – | – | – | – | – |
|  | 10. Anti-ARS | | – | – | + | – | – | + | + | + | + | + |
|  |  | Anti-Jo-1 | – | – | – | – | – | – | + | + | – | – |
|  |  | Anti-PL-7 | – | – | – | – | – | + | – | – | – | – |
|  |  | Anti-PL-12 | – | – | + | – | – | – | – | – | – | – |
|  |  | Anti-EJ | – | – | – | – | – | – | – | – | – | + |
|  |  | Anti-OJ | – | – | – | – | – | – | – | – | – | – |
|  |  | Anti-KS | – | – | – | – | – | – | – | – | + | – |
|  | 11. Anti-PM-Scl | | – | – | – | – | – | – | – | – | – | – |
|  | 12. Anti-MDA5 | | – | – | – | – | – | – | – | – | – | – |
| Morphological Domain | | | + | + | + | + | – | + | – | + | + | + |
|  | 1. High-resolution computed tomography | | + | + | + | + | – | + | – | + | + | + |
|  |  | a. Nonspecific interstitial pneumonia | + | + | + | + | – | + | – | + | + | + |
|  |  | b. Organizing pneumonia | – | – | – | – | – | – | – | – | – | – |
|  |  | c. Nonspecific interstitial pneumonia with organizing pneumonia | – | – | – | – | – | – | – | – | – | – |
|  |  | d. Lymphoid interstitial pneumonia | – | – | – | – | – | – | – | – | – | – |
|  |  | e. Interstitial lymphoid aggregates with germinal centers | – | – | – | – | – | – | – | – | – | – |
|  |  | f. Diffuse lymphoplasmacytic infiltration | – | – | – | – | – | – | – | – | – | – |
|  | 2. Multi-compartment involvement | | – | – | – | – | – | – | – | – | – | – |
|  |  | a. Unexplained pleural Effusion or thickening | – | – | – | – | – | – | – | – | – | – |
|  |  | b. Unexplained pericardial effusion or thickening | – | – | – | – | – | – | – | – | – | – |
|  |  | c. Unexplained intrinsic airways disease | – | – | – | – | – | – | – | – | – | – |
|  |  | d. Unexplained pulmonary vasculopathy | – | – | – | – | – | – | – | – | – | – |
| n/a: not applicable | | | | | | | | | | | | |
| Case 5 was classified as usual interstitial pneumonia and case 7 was classified as others in high-resolution computed tomography. | | | | | | | | | | | | |

**Supplementary Table S2. Characteristics and each domain of idiopathic interstitial pneumonia (IIP) patients who fulfilled interstitial pneumonia with autoimmune features (IPAF) criteria in the absence of anti-Ro52/tripartite motif-containing 21 (TRIM21) antibodies (anti-Ro52)**

| Case | |  | 1 | 2 | 3 | 4 | 5 | 6 | 7 | 8 | 9 | 10 |
| --- | --- | --- | --- | --- | --- | --- | --- | --- | --- | --- | --- | --- |
| Age (years) | | | 70 | 78 | 73 | 82 | 59 | 73 | 64 | 74 | 37 | 67 |
| Sex | |  | M | M | M | F | M | F | M | F | F | M |
| Clinical Domain | | | – | – | – | – | + | – | – | + | – | – |
|  | 1. Mechanic's hands | | – | – | – | – | n/a | – | – | – | – | – |
|  | 2. Distal digital tip ulceration | | – | – | – | – | + | – | – | – | – | – |
|  | 3. Inflammatory arthritis | | – | – | – | – | n/a | – | – | – | – | – |
|  | 4. Palmar telangiectasia | | – | – | – | – | n/a | – | – | – | – | – |
|  | 5. Raynaud's phenomenon | | – | – | – | – | n/a | – | – | + | – | – |
|  | 6. Unexplained digital oedema | | – | – | – | – | n/a | – | – | – | – | – |
|  | 7. Gottron's sign | | – | – | – | – | – | – | – | – | – | – |
| Serological Domain | | | + | + | + | + | – | + | + | + | + | + |
|  | 1. ANA positive | | – | – | – | – | – | – | – | + | – | + |
|  | ANA titer ≥ 1:320, diffuse, speckled, or homogeneous patterns | | – | – | – | – | – | – | – | + | – | + |
|  | ANA any titer, nucleolar or centromere patterns | | – | – | – | – | – | – | – | – | – | – |
|  | 2. Rheumatoid factor ≥ 2× upper limit of normal | | + | – | – | + | – | – | – | – | – | + |
|  | 3. Anti-CCP | | – | n/a | + | – | n/a | – | n/a | – | + | – |
|  | 4. Anti-double stranded DNA | | – | – | – | – | – | – | + | – | – | – |
|  | 5. Anti-Ro60/SS-A | | – | + | – | – | – | + | – | – | – | – |
|  | 6. Anti-La/SS-B | | – | + | – | – | – | – | – | – | – | – |
|  | 7. Anti-U1RNP | | – | – | – | – | – | – | – | – | + | – |
|  | 8. Anti-Sm | | – | – | – | – | – | – | – | – | – | – |
|  | 9. Anti-topoisomerase I (Scl-70) | | – | – | – | – | – | – | – | – | – | – |
|  | 10. Anti-ARS | | – | – | – | – | – | – | + | – | – | – |
|  |  | Anti-Jo-1 | – | – | – | – | – | – | – | – | – | – |
|  |  | Anti-PL-7 | – | – | – | – | – | – | – | – | – | – |
|  |  | Anti-PL-12 | – | – | – | – | – | – | – | – | – | – |
|  |  | Anti-EJ | – | – | – | – | – | – | + | – | – | – |
|  |  | Anti-OJ | – | – | – | – | – | – | – | – | – | – |
|  |  | Anti-KS | – | – | – | – | – | – | – | – | – | – |
|  | 11. Anti-PM-Scl | | – | – | – | – | – | – | – | – | – | – |
|  | 12. Anti-MDA5 | | – | – | – | – | – | – | – | – | – | – |
| Morphological Domain | | | + | + | + | + | + | + | + | – | + | + |
|  | 1. High-resolution computed tomography | | + | + | + | + | – | + | + | – | + | + |
|  |  | a. Nonspecific interstitial pneumonia | – | + | – | – | – | + | + | – | + | + |
|  |  | b. Organizing pneumonia | – | – | + | – | – | – | – | – | – | – |
|  |  | c. Nonspecific interstitial pneumonia with organizing pneumonia | – | – | – | – | – | – | – | – | – | – |
|  |  | d. Lymphoid interstitial pneumonia | – | – | – | – | – | – | – | – | – | – |
|  |  | e. Interstitial lymphoid aggregates with germinal centers | – | – | – | – | – | – | – | – | – | – |
|  |  | f. Diffuse lymphoplasmacytic infiltration | – | – | – | – | – | – | – | – | – | – |
|  | 2. Multi-compartment involvement | | + | – | – | – | + | – | – | – | – | – |
|  |  | a. Unexplained pleural Effusion or thickening | + | – | – | – | + | – | – | – | – | – |
|  |  | b. Unexplained pericardial effusion or thickening | – | – | – | – | – | – | – | – | – | – |
|  |  | c. Unexplained intrinsic airways disease | – | – | – | – | – | – | – | – | – | – |
|  |  | d. Unexplained pulmonary vasculopathy | – | – | – | – | – | – | – | – | – | – |
| n/a: not applicable | | | | | | | | | | | | |
| Case 5 was classified as diffuse alveolar damage and case 8 was classified as others in high-resolution computed tomography. | | | | | | | | | | | | |

(Continued)

| Case | |  | 11 | 12 | 13 | 14 | 15 | 16 | 17 | 18 | 19 | 20 |
| --- | --- | --- | --- | --- | --- | --- | --- | --- | --- | --- | --- | --- |
| Age (years) | | | 64 | 53 | 68 | 69 | 48 | 71 | 70 | 75 | 72 | 77 |
| Sex | |  | F | M | M | F | F | M | F | M | F | F |
| Clinical Domain | | | – | – | + | + | + | + | + | – | – | – |
|  | 1. Mechanic's hands | | – | – | – | – | – | – | – | – | – | – |
|  | 2. Distal digital tip ulceration | | – | – | – | – | – | – | – | – | – | – |
|  | 3. Inflammatory arthritis | | – | – | – | + | + | + | + | – | – | – |
|  | 4. Palmar telangiectasia | | – | – | + | – | – | – | – | – | – | – |
|  | 5. Raynaud's phenomenon | | – | – | – | – | – | – | – | – | – | – |
|  | 6. Unexplained digital oedema | | – | – | + | – | – | – | – | – | – | – |
|  | 7. Gottron's sign | | – | – | – | – | – | – | – | – | – | – |
| Serological Domain | | | + | + | + | + | – | + | + | + | + | + |
|  | 1. ANA positive | | – | – | – | + | – | + | – | – | – | – |
|  | ANA titer ≥ 1:320, diffuse, speckled, or homogeneous patterns | | – | – | – | + | – | + | – | – | – | – |
|  | ANA any titer, nucleolar or centromere patterns | | – | – | – | + | – | + | – | – | – | – |
|  | 2. Rheumatoid factor ≥ 2× upper limit of normal | | + | + | – | – | – | – | – | + | – | + |
|  | 3. Anti-CCP | | – | – | + | – | – | + | – | – | n/a | – |
|  | 4. Anti-double stranded DNA | | – | – | – | – | – | – | – | – | + | – |
|  | 5. Anti-Ro60/SS-A | | – | – | – | – | – | – | + | – | – | – |
|  | 6. Anti-La/SS-B | | – | – | – | – | – | – | – | – | – | – |
|  | 7. Anti-U1RNP | | – | – | – | – | – | – | – | – | – | – |
|  | 8. Anti-Sm | | – | – | – | – | – | – | – | – | – | – |
|  | 9. Anti-topoisomerase I (Scl-70) | | – | – | – | – | – | – | + | – | – | – |
|  | 10. Anti-ARS | | – | – | + | – | – | – | – | – | – | – |
|  |  | Anti-Jo-1 | – | – | – | – | – | – | – | – | – | – |
|  |  | Anti-PL-7 | – | – | – | – | – | – | – | – | – | – |
|  |  | Anti-PL-12 | – | – | – | – | – | – | – | – | – | – |
|  |  | Anti-EJ | – | – | + | – | – | – | – | – | – | – |
|  |  | Anti-OJ | – | – | – | – | – | – | – | – | – | – |
|  |  | Anti-KS | – | – | – | – | – | – | – | – | – | – |
|  | 11. Anti-PM-Scl | | – | – | – | – | – | – | – | – | – | – |
|  | 12. Anti-MDA5 | | – | – | – | – | – | – | – | – | – | – |
| Morphological Domain | | | + | + | – | – | + | – | – | + | + | + |
|  | 1. High-resolution computed tomography | | + | + | – | – | + | – | – | + | + | + |
|  |  | a. Nonspecific interstitial pneumonia | – | + | – | – | + | – | – | + | + | + |
|  |  | b. Organizing pneumonia | + | – | – | – | – | – | – | – | – | – |
|  |  | c. Nonspecific interstitial pneumonia with organizing pneumonia | – | – | – | – | – | – | – | – | – | – |
|  |  | d. Lymphoid interstitial pneumonia | – | – | – | – | – | – | – | – | – | – |
|  |  | e. Interstitial lymphoid aggregates with germinal centers | – | – | – | – | – | – | – | – | – | – |
|  |  | f. Diffuse lymphoplasmacytic infiltration | – | – | – | – | – | – | – | – | – | – |
|  | 2. Multi-compartment involvement | | – | – | – | – | – | – | – | – | – | – |
|  |  | a. Unexplained pleural Effusion or thickening | – | – | – | – | – | – | – | – | – | – |
|  |  | b. Unexplained pericardial effusion or thickening | – | – | – | – | – | – | – | – | – | – |
|  |  | c. Unexplained intrinsic airways disease | – | – | – | – | – | – | – | – | – | – |
|  |  | d. Unexplained pulmonary vasculopathy | – | – | – | – | – | – | – | – | – | – |
| n/a: not applicable | | | | | | | | | | | | |
| Case 13, 16 and 17 were classified as usual interstitial pneumonia and case 14 was classified as others in high-resolution computed tomography. | | | | | | | | | | | | |

(Continued)

| Case | |  | 21 | 22 | 23 | 24 | 25 | 26 | 27 | 28 | 29 | 30 |
| --- | --- | --- | --- | --- | --- | --- | --- | --- | --- | --- | --- | --- |
| Age (years) | | | 71 | 81 | 84 | 68 | 49 | 72 | 65 | 61 | 61 | 72 |
| Sex | |  | M | F | M | F | F | M | M | F | M | F |
| Clinical Domain | | | + | – | – | – | – | + | – | + | – | – |
|  | 1. Mechanic's hands | | – | – | – | – | – | – | – | – | – | – |
|  | 2. Distal digital tip ulceration | | + | – | – | – | – | – | – | – | – | – |
|  | 3. Inflammatory arthritis | | – | – | – | – | – | + | – | – | – | – |
|  | 4. Palmar telangiectasia | | – | – | – | – | – | – | – | – | – | – |
|  | 5. Raynaud's phenomenon | | + | – | – | – | – | – | – | – | – | – |
|  | 6. Unexplained digital oedema | | – | – | – | – | – | – | – | + | – | – |
|  | 7. Gottron's sign | | – | – | – | – | – | – | – | – | – | – |
| Serological Domain | | | + | + | + | + | + | – | + | + | + | + |
|  | 1. ANA positive | | – | – | – | + | – | – | + | – | – | – |
|  | ANA titer ≥ 1:320, diffuse, speckled, or homogeneous patterns | | – | – | – | – | – | – | + | – | – | – |
|  | ANA any titer, nucleolar or centromere patterns | | – | – | – | + | – | – | – | – | – | – |
|  | 2. Rheumatoid factor ≥ 2× upper limit of normal | | n/a | n/a | – | – | – | – | – | + | – | + |
|  | 3. Anti-CCP | | n/a | n/a | n/a | n/a | n/a | n/a | n/a | – | – | n/a |
|  | 4. Anti-double stranded DNA | | – | – | n/a | – | n/a | – | n/a | n/a | – | n/a |
|  | 5. Anti-Ro60/SS-A | | + | – | – | – | – | – | – | – | – | – |
|  | 6. Anti-La/SS-B | | – | – | – | – | – | – | – | – | – | – |
|  | 7. Anti-U1RNP | | – | – | – | – | – | – | – | – | – | – |
|  | 8. Anti-Sm | | – | – | – | – | – | – | – | – | – | – |
|  | 9. Anti-topoisomerase I (Scl-70) | | – | – | – | – | – | – | – | – | – | – |
|  | 10. Anti-ARS | | – | + | – | – | + | – | – | – | – | – |
|  |  | Anti-Jo-1 | – | – | – | – | + | – | – | – | – | – |
|  |  | Anti-PL-7 | – | + | – | – | – | – | – | – | – | – |
|  |  | Anti-PL-12 | – | – | – | – | – | – | – | – | – | – |
|  |  | Anti-EJ | – | – | – | – | – | – | – | – | – | – |
|  |  | Anti-OJ | – | – | – | – | – | – | – | – | – | – |
|  |  | Anti-KS | – | – | – | – | – | – | – | – | – | – |
|  | 11. Anti-PM-Scl | | – | – | – | – | – | – | – | – | – | – |
|  | 12. Anti-MDA5 | | – | – | – | – | – | – | – | – | – | – |
| Morphological Domain | | | – | + | + | + | + | + | + | + | + | + |
|  | 1. High-resolution computed tomography | | – | – | – | + | + | + | + | + | + | + |
|  |  | a. Nonspecific interstitial pneumonia | – | – | – | – | + | – | – | – | + | + |
|  |  | b. Organizing pneumonia | – | – | – | + | – | + | + | + | – | – |
|  |  | c. Nonspecific interstitial pneumonia with organizing pneumonia | – | – | – | – | – | – | – | – | – | – |
|  |  | d. Lymphoid interstitial pneumonia | – | – | – | – | – | – | – | – | – | – |
|  |  | e. Interstitial lymphoid aggregates with germinal centers | – | – | – | – | – | – | – | – | – | – |
|  |  | f. Diffuse lymphoplasmacytic infiltration | – | – | – | – | – | – | – | – | – | – |
|  | 2. Multi-compartment involvement | | – | + | + | + | – | – | – | – | – | – |
|  |  | a. Unexplained pleural Effusion or thickening | – | + | + | + | – | – | – | – | – | – |
|  |  | b. Unexplained pericardial effusion or thickening | – | – | – | – | – | – | – | – | – | – |
|  |  | c. Unexplained intrinsic airways disease | – | – | – | – | – | – | – | – | – | – |
|  |  | d. Unexplained pulmonary vasculopathy | – | – | – | – | – | – | – | – | – | – |
| n/a: not applicable | | | | | | | | | | | | |
| Case 22 and 23 were classified as diffuse alveolar damage and case 21 was classified as others in high-resolution computed tomography. | | | | | | | | | | | | |

(Continued)

| Case | |  | 31 | 32 | 33 | 34 | 35 | 36 | 37 | 38 | 39 | 40 |
| --- | --- | --- | --- | --- | --- | --- | --- | --- | --- | --- | --- | --- |
| Age (years) | | | 78 | 90 | 73 | 73 | 65 | 84 | 77 | 64 | 58 | 61 |
| Sex | |  | F | F | M | M | M | F | F | F | F | M |
| Clinical Domain | | | – | – | – | – | – | – | + | – | + | – |
|  | 1. Mechanic's hands | | – | – | – | – | – | – | – | – | – | – |
|  | 2. Distal digital tip ulceration | | – | – | – | – | – | – | – | – | – | – |
|  | 3. Inflammatory arthritis | | – | – | – | – | – | – | + | – | – | – |
|  | 4. Palmar telangiectasia | | – | – | – | – | – | – | – | – | – | – |
|  | 5. Raynaud's phenomenon | | – | – | – | – | – | – | – | – | + | – |
|  | 6. Unexplained digital oedema | | – | – | – | – | – | – | – | – | – | – |
|  | 7. Gottron's sign | | – | – | – | – | – | – | – | – | – | – |
| Serological Domain | | | + | + | + | + | + | + | + | + | – | + |
|  | 1. ANA positive | | – | + | – | – | + | – | – | – | – | – |
|  | ANA titer ≥ 1:320, diffuse, speckled, or homogeneous patterns | | – | + | – | – | + | – | – | – | – | – |
|  | ANA any titer, nucleolar or centromere patterns | | – | – | – | – | – | – | – | – | – | – |
|  | 2. Rheumatoid factor ≥ 2× upper limit of normal | | – | – | – | + | – | – | – | + | – | + |
|  | 3. Anti-CCP | | n/a | – | + | n/a | – | – | + | n/a | n/a | n/a |
|  | 4. Anti-double stranded DNA | | – | – | – | n/a | + | + | – | n/a | n/a | n/a |
|  | 5. Anti-Ro60/SS-A | | – | – | – | – | – | – | – | – | – | – |
|  | 6. Anti-La/SS-B | | – | – | – | – | – | – | – | – | – | – |
|  | 7. Anti-U1RNP | | – | – | – | – | – | – | – | – | – | – |
|  | 8. Anti-Sm | | – | – | – | – | – | – | – | – | – | – |
|  | 9. Anti-topoisomerase I (Scl-70) | | – | – | – | – | – | – | – | – | – | – |
|  | 10. Anti-ARS | | + | – | – | – | – | – | – | – | – | – |
|  |  | Anti-Jo-1 | – | – | – | – | – | – | – | – | – | – |
|  |  | Anti-PL-7 | – | – | – | – | – | – | – | – | – | – |
|  |  | Anti-PL-12 | – | – | – | – | – | – | – | – | – | – |
|  |  | Anti-EJ | + | – | – | – | – | – | – | – | – | – |
|  |  | Anti-OJ | – | – | – | – | – | – | – | – | – | – |
|  |  | Anti-KS | – | – | – | – | – | – | – | – | – | – |
|  | 11. Anti-PM-Scl | | – | – | – | – | – | – | – | – | – | – |
|  | 12. Anti-MDA5 | | – | – | – | – | – | – | – | – | – | – |
| Morphological Domain | | | + | + | + | + | + | + | + | + | + | + |
|  | 1. High-resolution computed tomography | | + | + | + | + | + | + | + | + | + | + |
|  |  | a. Nonspecific interstitial pneumonia | + | – | + | + | + | + | + | + | + | + |
|  |  | b. Organizing pneumonia | – | – | – | – | – | – | – | – | – | – |
|  |  | c. Nonspecific interstitial pneumonia with organizing pneumonia | – | + | – | – | – | – | – | – | – | – |
|  |  | d. Lymphoid interstitial pneumonia | – | – | – | – | – | – | – | – | – | – |
|  |  | e. Interstitial lymphoid aggregates with germinal centers | – | – | – | – | – | – | – | – | – | – |
|  |  | f. Diffuse lymphoplasmacytic infiltration | – | – | – | – | – | – | – | – | – | – |
|  | 2. Multi-compartment involvement | | – | + | – | – | – | – | – | – | – | – |
|  |  | a. Unexplained pleural Effusion or thickening | – | + | – | – | – | – | – | – | – | – |
|  |  | b. Unexplained pericardial effusion or thickening | – | – | – | – | – | – | – | – | – | – |
|  |  | c. Unexplained intrinsic airways disease | – | – | – | – | – | – | – | – | – | – |
|  |  | d. Unexplained pulmonary vasculopathy | – | – | – | – | – | – | – | – | – | – |
| n/a: not applicable | | | | | | | | | | | | |

(Continued)

| Case | |  | 41 | 42 | 43 | 44 | 45 |
| --- | --- | --- | --- | --- | --- | --- | --- |
| Age (years) | | | 70 | 62 | 54 | 72 | 66 |
| Sex | |  | F | M | F | F | F |
| Clinical Domain | | | – | + | + | – | – |
|  | 1. Mechanic's hands | | – | + | + | – | – |
|  | 2. Distal digital tip ulceration | | – | – | – | – | – |
|  | 3. Inflammatory arthritis | | – | – | – | – | – |
|  | 4. Palmar telangiectasia | | – | – | – | – | – |
|  | 5. Raynaud's phenomenon | | – | – | – | – | – |
|  | 6. Unexplained digital oedema | | – | – | – | – | – |
|  | 7. Gottron's sign | | – | – | – | – | – |
| Serological Domain | | | + | + | – | + | + |
|  | 1. ANA positive | | + | – | – | – | + |
|  | ANA titer ≥ 1:320, diffuse, speckled, or homogeneous patterns | | – | – | – | – | + |
|  | ANA any titer, nucleolar or centromere patterns | | + | – | – | – | + |
|  | 2. Rheumatoid factor ≥ 2× upper limit of normal | | n/a | + | – | – | n/a |
|  | 3. Anti-CCP | | – | – | – | – | – |
|  | 4. Anti-double stranded DNA | | n/a | – | – | – | – |
|  | 5. Anti-Ro60/SS-A | | – | – | – | – | + |
|  | 6. Anti-La/SS-B | | – | – | – | – | – |
|  | 7. Anti-U1RNP | | – | – | – | – | – |
|  | 8. Anti-Sm | | – | – | – | – | – |
|  | 9. Anti-topoisomerase I (Scl-70) | | – | – | – | – | – |
|  | 10. Anti-ARS | | – | + | – | – | – |
|  |  | Anti-Jo-1 | – | – | – | – | – |
|  |  | Anti-PL-7 | – | – | – | – | – |
|  |  | Anti-PL-12 | – | – | – | – | – |
|  |  | Anti-EJ | – | – | – | – | – |
|  |  | Anti-OJ | – | – | – | – | – |
|  |  | Anti-KS | – | + | – | – | – |
|  | 11. Anti-PM-Scl | | – | – | – | – | – |
|  | 12. Anti-MDA5 | | – | – | – | + | – |
| Morphological Domain | | | + | + | + | + | + |
|  | 1. High-resolution computed tomography | | + | + | + | + | + |
|  |  | a. Nonspecific interstitial pneumonia | + | + | + | + | + |
|  |  | b. Organizing pneumonia | – | – | – | – | – |
|  |  | c. Nonspecific interstitial pneumonia with organizing pneumonia | – | – | – | – | – |
|  |  | d. Lymphoid interstitial pneumonia | – | – | – | – | – |
|  |  | e. Interstitial lymphoid aggregates with germinal centers | – | – | – | – | – |
|  |  | f. Diffuse lymphoplasmacytic infiltration | – | – | – | – | – |
|  | 2. Multi-compartment involvement | | – | – | – | – | – |
|  |  | a. Unexplained pleural Effusion or thickening | – | – | – | – | – |
|  |  | b. Unexplained pericardial effusion or thickening | – | – | – | – | – |
|  |  | c. Unexplained intrinsic airways disease | – | – | – | – | – |
|  |  | d. Unexplained pulmonary vasculopathy | – | – | – | – | – |
| n/a: not applicable | | |  |  |  |  |  |

**Supplementary Table S3. Clinical characteristics of the anti-aminoacyl-tRNA synthetases antibodies (anti-ARS) positive patients in the presence or absence of anti-Ro52/tripartite motif-containing 21 (TRIM21) antibodies (anti-Ro52)**

|  |  | All | Anti-Ro52 positive | Anti-Ro52 negative | *P* value |
| --- | --- | --- | --- | --- | --- |
| Subjects | | n = 18 | n = 8 | n = 10 | – |
| Age (years) | | 64 [62–69] | 64 [59–65] | 66 [62–76] | 0.35 |
| Male, n (%) | | 11 (61) | 4 (50%) | 7 (70) | 0.35 |
| Smoking (Pack-years) | | 21 [0–66] | 22 [0–42] | 22 [0–72] | 0.67 |
| Fulfilled IPAF criteria, n (%) | | 12 (67) | 6 (75) | 6 (60) | 0.44 |
|  | Clinical Domain, n (%) | 4 (22) | 2 (25) | 2 (20) | 0.62 |
|  | Serological Domain, n (%) | 18 (100) | 8 (100) | 10 (100) | – |
|  | Morphological Domain, n (%) | 10 (56) | 5 (63) | 5 (50) | 0.48 |
| Respiratory Symptoms | |  |  |  |  |
|  | Cough, n (%) | 11 (65) | 6 (75) | 5 (56) | 0.37 |
|  | Sputum^#^, n (%) | 0 (0) | 0 (0) | 0 (0) | – |
|  | Dypnea^#^, n (%) | 15 (83) | 7 (88) | 8 (80) | 0.59 |
| Clinical symptoms related to SARD | |  |  |  |  |
|  | Mechanic's hands, n (%) | 2 (11) | 1 (13) | 1 (10) | 0.71 |
|  | Distal digital tip ulceration, n (%) | 0 (0) | 0 (0) | 0 (0) | – |
|  | Inflammatory arthritis, n (%) | 1 (6) | 1 (13) | 0 (0) | 0.44 |
|  | Palmar telangiectasia, n (%) | 1 (6) | 0 (0) | 1 (10) | – |
|  | Raynaud's phenomenon, n (%) | 1 (6) | 1 (13) | 0 (0) | 0.44 |
|  | Unexplained digital oedema, n (%) | 1 (6) | 0 (0) | 1 (10) | 0.56 |
|  | Gottron's sign, n (%) | 0 (0) | 0 (0) | 0 (0) | – |
|  | Muscle weakness, n (%) | 0 (0) | 0 (0) | 0 (0) | – |
|  | Weight loss, n (%) | 2 (11) | 1 (13) | 1 (10) | 0.71 |
|  | Dry mouth or dry eye, n (%) | 0 (0) | 0 (0) | 0 (0) | – |
|  | Dysphagia, n (%) | 0 (0) | 0 (0) | 0 (0) | – |
|  | Gastroesophageal reflux disease^¶^, n (%) | 0 (0) | 0 (0) | 0 (0) | – |
| Data presented as median [interquartile range] or frequencies (%). | | | | | |
| n=18, unless otherwise stated; ^#^: n=17; ^¶^: n=4 | | | | | |
| Ro52: Ro52/tripartite motif-containing 21; IPAF: interstitial pneumonia with autoimmune features; CTD: connective tissue disease | | | | | |
| *P* value: anti-Ro52 positive vs. anti-Ro52 negative | | | | | |

**Supplementary Table S4. Laboratory tests, pulmonary function test and bronchoalveolar lavage fluid findings of the anti-aminoacyl-tRNA synthetases antibodies (anti-ARS) positive patients in the presence or absence of anti-Ro52/tripartite motif-containing 21 (TRIM21) antibodies (anti-Ro52)**

|  |  | All | Anti-Ro52 positive | Anti-Ro52 negative | *P* value |
| --- | --- | --- | --- | --- | --- |
| Subjects | | n = 18 | n = 8 | n = 10 | – |
| Laboratory findings | |  |  |  |  |
|  | CRP (mg/dL) | 0.35 [0.15–3.63] | 0.21 [0.17–1.35] | 0.54 [0.14–11.6] | 0.62 |
|  | LDH (IU/L)^#^ | 256 [196–348] | 317 [187–221] | 404 [209–293] | 0.29 |
|  | CK (IU/L)^¶^ | 80 [66–145] | 136 [80–136] | 70 [60–137] | 0.24 |
|  | KL-6 (U/mL)^+^ | 1585 [900–1843] | 1130 [807–1710] | 1611 [1294–2264] | 0.45 |
|  | SP-A (ng/mL)^§^ | 81 [55–103] | 71 [47–110] | 81 [65–103] | 0.81 |
|  | SP-D (ng/mL)^ƒ^ | 155 [109–310] | 145 [126–151] | 308 [94–345] | 0.26 |
| Pulmonary function tests | |  |  |  |  |
|  | VC (% predicted) | 63 [58–73] | 65 [56–77] | 62 [60–71] | 0.80 |
|  | FEV_1_/FEV (% predicted) | 79 [74–87] | 83 [77–90] | 78 [72–83] | 0.37 |
|  | DL_CO_ (% predicted) | 49 [36–59] | 40 [32–58] | 56 [47–62] | 0.32 |
| Bronchoalveolar lavage fluid^§^ | |  |  |  |  |
|  | TCC (×10^5^/mL) | 3.3 [2.4–5.9] | 4.4 [2.4–7.5] | 3.3 [2.4–4.2] | 0.81 |
|  | Macrophages (%) | 51.8 [45.0–60.1] | 61.5 [35.6–61.4] | 51.8 [49.2–59.4] | 0.62 |
|  | Lymphocytes (%) | 20.4 [13.5–33.4] | 13.1 [7.2–42.2] | 28.1 [20.4–33.4] | 0.22 |
|  | Neutrophils (%) | 8.4 [4.0–16.9] | 4.0 [2.7–28.1] | 10.9 [8.4–16.9] | 0.22 |
|  | Eosinophils (%) | 4.0 [2.8–6.6] | 2.6 [1.4–3.8] | 6.6 [6.4–9.1] | 0.050 |
|  | CD4/CD8 | 0.4 [0.2–0.6] | 0.4 [0.1–1.4] | 0.4 [0.2–0.4] | 0.81 |
| Data presented as median [interquartile range] or frequencies (%). | | | | | |
| n=18, unless otherwise stated; ^#^: n=16; ^¶^: n=13; ^+^: n=17; ^§^: n=9; ^ƒ^: n=11 | | | | | |
| Ro52: Ro52/tripartite motif-containing 21; CRP: C-reactive protein; LDH: lactate dehydrogenase; CK: creatine kinase; KL-6: Krebs von den Lungen-6; SP-A: surfactant protein-A; SP-D: surfactant protein-D; VC: vital capacity; FEV_1_: forced expiratory volume in one second; DL_CO_: diffusing capacity of the lung for carbon monoxide; TCC: total cell counts; CD: cluster of differentiation | | | | | |
|
|
| *P* value: anti-Ro52 positive vs. anti-Ro52 negative | | | | | |

**Supplementary Table S5. HRCT findings and patterns of the anti-aminoacyl-tRNA synthetases antibodies (anti-ARS) positive patients in the presence or absence of anti-Ro52/tripartite motif-containing 21 (TRIM21) antibodies (anti-Ro52)**

|  |  | All | Anti-Ro52 positive | Anti-Ro52 negative | *P* value |
| --- | --- | --- | --- | --- | --- |
| Subjects | | n = 18  n (%) | n = 8  n (%) | n = 10  n (%) | – |
| HRCT findings | |  |  |  |  |
|  | Volume loss | 17 (94) | 7 (88) | 10 (100) | 0.44 |
|  | Lower distribution | 17 (94) | 8 (100) | 9 (90) | 0.56 |
|  | Subpleural distribution | 11 (61) | 4 (50) | 7 (70) | 0.35 |
|  | Peribronchial distribution | 4 (22) | 1 (13) | 3 (30) | 0.38 |
|  | Reticular shadow | 17 (94) | 7 (88) | 10 (100) | 0.44 |
|  | Honeycombing | 4 (22) | 3 (38) | 1 (10) | 0.21 |
|  | Traction bronchiectasis | 17 (94) | 7 (88) | 10 (100) | 0.44 |
|  | Ground-glass attenuation | 13 (72) | 5 (63) | 8 (80) | 0.38 |
|  | Consolidation | 6 (33) | 3 (38) | 3 (30) | 0.56 |
|  | Thickening of BVB | 1 (6) | 0 (0) | 1 (10) | 0.56 |
|  | Small nodules (φ < 5 mm) | 0 (0) | 0 (0) | 0 (0) | – |
|  | Nodules (φ > 5 mm) | 0 (0) | 0 (0) | 0 (0) | – |
|  | Pleural effusion | 1 (6) | 0 (0) | 1 (10) | 0.56 |
| HRCT pattern | |  |  |  |  |
|  | UIP | 4 (22) | 1 (13) | 3 (30) | 0.38 |
|  | NSIP | 10 (56) | 5 (63) | 5 (50) | 0.48 |
|  | OP | 0 (0) | 0 (0) | 0 (0) | – |
|  | DAD | 2 (11) | 0 (0) | 2 (20) | 0.29 |
|  | Others | 2 (11) | 2 (25) | 0 (0) | 0.18 |
| Data presented as frequencies (%). | | | | | |
| Ro52: Ro52/tripartite motif-containing 21; HRCT: high-resolution computed tomography; BVB: bronchovascular bundles; UIP: usual interstitial pneumonia; NSIP: nonspecific interstitial pneumonia; OP: organizing pneumonia; DAD: diffuse alveolar damage | | | | | |
|
| *P* value: anti-Ro52 positive vs. anti-Ro52 negative | | | | | |
